# Performance of a Quantitative PCR-Based Assay and Beta-d-Glucan Detection for Diagnosis of Invasive Candidiasis in Very-Low-Birth-Weight Preterm Neonatal Patients (CANDINEO Study)

**DOI:** 10.1128/JCM.00496-17

**Published:** 2017-08-23

**Authors:** Jose Tomas Ramos, Sonia Villar, Emilio Bouza, Elena Bergon-Sendin, Alfredo Perez Rivilla, Caridad Tapia Collados, Mariano Andreu, Candelaria Santana Reyes, María Isolina Campos-Herrero, Jon López de Heredia, María Cruz López Herrera, Paloma Anguita Alonso, Carmen Rosa Pallás-Alonso, Manuel Cuenca-Estrella, the CANDINEO Study Group

**Affiliations:** a Hospital Universitario Clínico San Carlos, Departamento de Pediatría, and Universidad Complutense, Instituto de Investigación Sanitaria del Hospital Clínico San Carlos (IdISSC), Madrid, Spain; b Hospital Materno-Infantil Gregorio Marañón, Servicio de Neonatología, Madrid, Spain; c Hospital General Universitario Gregorio Marañón CIBERES, IISGM Universidad Complutense de Madrid, Madrid, Spain; d Hospital Universitario 12 de Octubre, Servicio de Neonatología, Universidad Complutense, and Red de Salud Materno Infantil y del Desarrollo SAMID, Madrid, Spain; e Hospital Universitario 12 de Octubre, Servicio de Microbiología, Madrid, Spain; f Hospital General Universitario de Alicante, Servicio de Neonatología, Alicante, Spain; g Hospital General Universitario de Alicante, Servicio de Microbiología, Alicante, Spain; h Hospital Materno-Infantil Insular de Las Palmas, Servicio de Neonatología, Las Palmas de Gran Canaria, Spain; i Hospital Universitario de Gran Canaria Doctor Negrín, Hospital Materno-Infantil, Servicio de Microbiología, Las Palmas de Gran Canaria, Spain; j Hospital de Cruces, Servicio de Neonatología, Bilbao, Spain; k Astellas Pharma S.A., Madrid, Spain; l Instituto de Salud Carlos III, Majadahonda, Madrid, Spain

**Keywords:** invasive candidiasis, serum beta-d-glucan, PCR, polymerase chain reaction

## Abstract

An epidemiological, multicenter, noninterventional, observational case-control study was conducted to describe the performance of serum beta-d-glucan (BDG) and Candida PCR in blood, serum, and sterile samples for the diagnosis of invasive candidiasis (IC) in very-low-birth-weight (VLBW) preterm neonates and to compare these techniques with culture of samples from blood and other sterile sites. Seventeen centers participated in the study, and the number of episodes analyzed was 159. A total of 9 episodes of IC from 9 patients (7 confirmed and 2 probable) and 150 episodes of suspected sepsis from 117 controls were identified. The prevalence of IC was 5.7% (95% confidence interval [95% CI], 2.1 to 9.3). The mortality was significantly higher in episodes of IC (44.4%) than in the non-IC episodes (11.1%, *P* < 0.01). The sensitivity and specificity of the PCR performed on blood/serum samples were 87.5% and 81.6%, respectively. The sensitivity and specificity of the BDG results were lower (75.0% and 64.6%). For cases with negative culture results, the PCR and the BDG results were positive in 27 (17.4%) and 52 (33.5%) episodes, respectively. The presence of multiorgan failure, improvement with empirical antifungal therapy, thrombocytopenia, and Candida colonization were significantly associated (*P* < 0.01) with PCR or BDG positivity regardless of the results of the cultures. Serum BDG analysis and Candida PCR could be used as complementary diagnostic techniques to detect IC in VLBW neonates.

## INTRODUCTION

Candida species infection is one of the leading causes of death in very-low-birth-weight (VLWB; <1,250 g) preterm infants (1). Candida spp. represent the third-most-common pathogens isolated from bloodstream infections of preterm infants in neonatal intensive care units (NICU) and the fourth-most-common pathogens in pediatric intensive care unit patients (2, 3). Candida albicans is the most common fungal species infecting neonates, followed by Candida parapsilosis (1, 4). In addition, Candida species infections tend to disseminate as invasive candidiasis (IC) in VLWB neonates more frequently than in adults or older children throughout all organ systems, including the central nervous system (CNS), leading to a high mortality rate and neurocognitive impairment in survivors (1, 5, 6). The successful management of these patients requires the early detection and identification of the infectious agent to guide the initiation of appropriate antifungal therapy.

The prevalences of IC, which differ between individual NICUs and according to the presence of associated risk factors, range from 3% to as high as 23% in VLBW neonates (1). Although early treatment improves survival, beginning appropriate therapy is complicated because the clinical diagnosis of IC is very problematic due to the nonspecific signs of the infection and the 48 to 72 h required to obtain the results of microbiologic cultures (1, 6). Major challenges to the diagnosis of Candida species infections in the NICU are the difficulties in obtaining blood samples of sufficient volume and the low yield of blood cultures (6).

Analyses using serum levels of the beta-d-glucan (BDG) biomarker and nucleic acid identification detection by PCR-based techniques have shown promising results for the detection of IC in adults and older children at risk of the infection (7–13). However, there is scant information on the role of biomarkers and PCR in VLBW neonates (12–16), in whom an accurate and rapid diagnosis of Candida species infections in the NICU is of paramount importance. While BDG analyses may lack specificity in VLBW newborns who are colonized by Candida spp. (15, 16), PCR might be a good tool for the diagnosis of IC in these infants due to the low blood volume required for testing and its rapid turnaround time. PCR, using both whole-blood and serum samples, has shown good sensitivity and specificity in immunocompromised and adult intensive care unit (ICU) patients (7, 8), although very few studies (with small patient numbers) have been performed in neonates (13, 14).

This multicenter study aimed to describe the performance of serum BDG and Candida species PCR in blood, serum, and sterile samples for the diagnosis of IC in VLBW neonates in the NICU and to compare these techniques with culture of samples from blood and other sterile sites. This study also aimed to analyze the usefulness of BDG and PCR in episodes with negative Candida species cultures, to compare the epidemiological characteristics of patients with and without IC, and to describe the microbiological spectrum of Candida species isolates from VLBW (<1,250-g) preterm neonates admitted to the NICU.

## RESULTS

### Patient enrollment/episodes and characteristics of the patients analyzed.

Seventeen centers participated in the study, although 7 hospitals did not enroll any patients. The total number of episodes analyzed was 159, corresponding to 126 neonates who met the inclusion criteria and for whom informed consent was obtained. Of these, there were 99 patients with one episode, 21 with two episodes, and 6 with three episodes. Among the participating hospitals, 118 episodes (74.2%) were from four centers. The main patient characteristics of the study patients, including a summary of the key characteristics of the 126 neonates included in the study, are shown in Table 1, together with comparisons between the case and control groups. Data are presented for all patients and also according to IC or non-IC classifications.

**TABLE 1:** Characteristics of the study patients, with comparison of IC patients to non-IC patients^*a*^

Variable	Value(s)			*P* value
	All patients (*n* = 126)	IC patients (*n* = 9)	Non-IC patients (*n* = 117)	
Male sex, %	50.0	57.1	49.6	0.50
Mean age upon admission to the NICU, min (range)	1,898 (0–44,640)	2,753 (15–12,000)	1,848 (0–44,640)	0.72
Mean gestational age, days (range)	189 (168–224)	180 (168–196)	190 (168–224)	0.08
Mean birth ht, cm (range)	34.2 (26–41)	33.3 (29–37)	34.3 (26–41)	0.31
Mean birth wt, g (range)	881.7 (495–1,249)	794.7 (600–1,220)	886.8 (495–1,249)	0.70
Mean CRIB (range)	4.6 (0–13)	6.2 (2–8)	4.5 (0–13)	0.25
Premature membrane rupture, %	35.0	14.3	36.2	0.28
Rupture at <24 h, %	11.4	14.3	11.2	0.58
Intrapartum antibiotic, %	52.8	28.6	54.5	0.17

a Premature membrane rupture data included 18 cases due to chorioamnionitis and 23 due to causes unknown. IC, invasive candidiasis; CRIB, clinical risk index for babies.

The analysis was conducted with respect to both the number of patients and the number of episodes. Each episode was independently considered. A total of 9 samples representing episodes of IC from 9 patients and 150 samples representing episodes from 117 controls were collected. The nine cases of IC were classified as seven confirmed cases and two probable cases (positive urine cultures with additional clinical criteria as defined above). The distribution of the Candida spp. for the nine episodes of IC was as follows: eight C. albicans episodes and one episode caused by a Candida spp. different from the six species of Candida detected by the PCR-based assay.

The controls comprised cases of bacterial infection together with cases where IC was not detected. A total of 76 episodes of bacteremia and 71 episodes in which blood cultures were considered negative were identified. In addition, there were three episodes that were interpreted as representative of colonization, based on a positive Candida species culture without clinical signs or symptoms of infection. These three episodes of colonization were also considered controls in the analysis.

The prevalences of IC (confirmed and probable) were 5.7% (9 cases/159 episodes; 95% confidence interval [95% CI], 2.1% to 9.3%) in assessing the number of episodes and 7.1% (9 cases/126 patients; 95% CI, 2.6% to 11.6%) in assessing the number of patients.

The number of the reported episodes of bacteremia was 76 (47.8%). The most frequent bacteria involved in these episodes were coagulase-negative staphylococci (the clinical significance of these isolates was not specified) and Gram-negative bacilli. The distribution of bacteremia episodes is described in Table 2.

**TABLE 2:** Distribution of bacteremia episodes

Class of bacteremia	Episodes	
	*n*	%
Coagulase-negative staphylococci	52	32.7
Gram-negative bacilli	12	7.5
Enterococci	5	3.1
Staphylococcus aureus	2	1.2
Group B streptococci	2	1.2
Multibacterial	4	2.4
Total	76	47.8

### Analysis of possible risk factors, clinical characteristics, and laboratory parameters of IC episodes.

Table 3 summarizes the most relevant data regarding risk factors overall and in each group of episodes. No differences were found between IC and non-IC episodes with regard to age, gestational age, birth weight, premature rupture of obstetric membranes, or intrapartum antibiotics. The data represent the clinical risk index for babies (CRIB) score; the number of days of breastfeeding; or predisposing factors such as prior surgery, necrotizing enterocolitis, parenteral nutrition, the number of days of intravenous lipids, the presence of mechanical ventilation, or recent central venous catheter placement or administration of antibiotics or antacids. Prior Candida species cultures in nonsterile sites were reported for 127 episodes (79.7%). The episodes of IC were significantly more commonly associated with prior Candida species colonization (44.4% versus 2.8%, *P* < 0.01) or with candida dermatitis (55.6% versus 1.4%, *P* < 0.01). The non-IC episodes were significantly associated with a prolonged time of mechanical ventilation (175 h versus 39.4 h, *P* < 0.01). No other significant differences were observed among the clinical variables analyzed. No significant differences were observed in the analyzed laboratory parameters, except for metabolic acidosis. Significantly more IC episodes than non-IC episodes were associated with metabolic acidosis (44.4% versus 7.5%, *P* < 0.01). The significance was maintained when only the bacteremia controls were analyzed, even when those involving coagulase-negative staphylococci were excluded. There was also a trend for more hyperglycemia in episodes of IC (*P* = 0.058). Table 4 shows the results of the analysis of the laboratory variables.

**TABLE 3:** Possible baseline risk factors for IC in clinical episodes^*a*^

Variable	Value(s)			*P* value
	All episodes (*n* = 159)	IC episodes (*n* = 9)	Non-IC episodes (*n* = 150)	
Mean age when suspected of IC, days (range)	19.7 (4–96)	15 (4–37)	20 (4–96)	0.42
Mean wt when suspected of IC, g (range)	1,010.7 (465–1,740)	902.2 (500–1,740)	1,017.4 (465–1,640)	0.98
Mean age of the commencement of breastfeeding, days (range)	3 (0–11)	4.2 (2–9)	2.9 (0–11)	0.67
Mean age of the commencement of enteral nutrition, days (range)	2.4 (0–13)	3.4 (1–8)	2.4 (0–13)	0.49
Received parenteral nutrition in the previous 15 days, %	90.4	100	89.8	0.39
Mean duration of parenteral nutrition, days (range)	14.5 (1–432)	11.4 (4–25)	14.7 (1–432)	0.70
Received intravenous lipids in the previous 15 days, %	87.2	88.9	87.1	0.67
Mean duration of intravenous lipids, days (range)	14.1 (1–408)	11.1 (3–25)	14.3 (1–408)	0.76
Received mechanical ventilation in the previous 48 h, %	39	55.6	37.9	0.23
Mean duration of mechanical ventilation, h (range)	162.7 (5–960)	39.4 (5–48)	175 (8–960)	0.02
CPAP connection in the previous 48 h, %	59	66.7	58.5	0.45
Mean induration in CPAP, h (range)	154 (5–999)	101 (19–324)	158.5 (5–999)	0.30
Central catheter in the previous 48 h, %	63.5	66.7	63.3	0.57
Mean duration with central catheter, h (range)	152 (3–648)	115 (48–168)	154 (3–648)	0.09
Peripheral catheter in the previous 48 h, %	48.4	66.7	47.3	0.21
Mean duration with peripheral catheter, h (range)	123 (8–720)	106 (48–324)	124 (8–720)	0.40
Prior surgery in the previous 15 days, %	12.2	11.1	12.2	0.69
Gastrointestinal surgery, %	6.4	11.1	6.1	0.45
Other surgeries, %	6.4	0	6.8	0.60
Prior Candida colonization, %	5.2 (20.3% not done)	44.4 (22.2% not done)	2.8 (20.1% not done)	0.000
Candida dermatitis, %	4.6	55.6	1.4	0.000
Necrotizing enterocolitis, %	13.5	22.2	13	0.16
Antacids in the previous 15 days, %	8.4	0	9	0.44
Antibiotic in the previous 15 days, %	79.5	77.8	79.6	0.58
Prior antifungal, %	39.6	44.4	39.3	0.51

a CPAP, continuous positive airway pressure; IC, invasive candidiasis.

**TABLE 4:** Association of clinical characteristics with clinical episodes of IC^*a*^

Variable	Value(s)			*P* value
	All episodes (*n* = 159)	IC episodes (*n* = 9)	Non-IC episodes (*n* = 150)	
Fever (>38°C) or hypothermia (<36.5°C), %	43.8	50.0	43.5	0.49
Tachycardia (>200/min) or bradycardia, %	56.8	55.6	56.8	0.60
Metabolic acidosis (<10 mval/liter), %	9.7	44.4	7.5	0.001
Apnea (>20 s), %	61.3	66.7	61.0	0.51
Apathy, %	63.6	44.4	65.5	0.10
Recapillarization time (>2 s), %	23.4	22.2	23.4	0.50
Food intolerance, %	38.5	33.3	38.8	0.24
Higher oxygen supplementation, %	62.8	44.4	63.9	0.46
Hyperglycemia (>140 mg/dl), %	23.1	55.6	21.7	0.058
Mean no. of platelets at the beginning of the episode, cells/ml (range)	216,966 (5,000–764,000)	185,750 (48,000–511,000)	218,873 (5,000–764,000)	0.45
Mean levels of C-reactive protein (preinfection), mg/dl (range)	1.5 (0–29)	2.3 (0–13.8)	1.4 (0–29)	0.32
Mean levels of C-reactive protein (postinfection), mg/dl (range)	7.3 (0–100)	7.7 (0–26)	7.3 (0–100)	0.14
Mean no. of total leukocytes (preinfection), cells/ml (range)	15,868 (1,470–50,300)	19,600 (7,500–50,300)	15,515 (1,470–39,500)	0.31
Mean no. of total leukocytes (postinfection), cells/ml (range)	15,995 (1,400–57,800)	15,312 (7,000–21,600)	16,039 (1,400–57,800)	0.24

a IC, invasive candidiasis.

### Progression, complications, and mortality.

Antifungal treatments were administered for 33 episodes (20.8%) and consisted of the following: liposomal amphotericin B (*n* = 29), micafungin (*n* = 2), fluconazole (*n* = 1), and fluconazole plus amphotericin B (*n* = 1). Empirical therapy was used in 23 episodes, and the treatment used for 10 episodes was considered to have been guided on the basis of clinical and radiological criteria, along with positive cultures. No patient received antifungal prophylaxis prior to empirical antifungal therapy. In 146 of 156 episodes (93.6%), empirical antibacterial therapy was also given simultaneously. The duration of antifungal treatment was significantly greater in episodes of IC than in non-IC episodes (19.1 versus 9.3 days, *P* < 0.01).

Even with the small number of IC episodes, the mortality was significantly higher in the episodes of IC than in the non-IC episodes. Overall, 17 patients died, which corresponded to total mortality rates of 10.7% (17/159) of episodes and 13.5% of patients (17/126). Among the 9 patients with IC, 4 died (44.4%), compared with 13 of 117 non-IC patients (11.1%) (*P* < 0.01). Moreover, significantly more IC patients with episodes had multiorgan failure (44.4% versus 6.8%, *P* < 0.01). Table 5 shows the outcome and mortality of patients with IC episodes compared with those of patients with non-IC episodes.

**TABLE 5:** Outcome of IC episodes and non-IC episodes^*a*^

Variable	Value(s)			*P* value
	Total patients (*n* = 126)	IC patients (*n* = 9)	Non-IC patients (*n* = 117)	
Death, %	13.7	44.4	12.0	0.01
Multiorgan failure, %	9.0	44.4	6.8	0.00
Mean time to episode resolution, days (range)	30.78 (5–121)	40.6 (13–79)	30.2 (5–121)	0.10

a IC, invasive candidiasis.

### Diagnostic tests for IC.

Most patients (>95%) underwent extensive testing that included imaging techniques and microbiological cultures for sterile and nonsterile sites as shown in Table 6. According to the clinical data and the results of the cultures, the episodes were classified as confirmed IC or probable IC. Of the nine episodes of IC, the blood culture results were positive in three and sterile fluid or peritoneum results were positive after surgery in four, and urine culture was positive along with improvement with antifungal therapy in the two patients with probable IC.

**TABLE 6:** Diagnostic tests and microbiologic cultures performed in the study patients

Diagnostic test	No. (%) performed (*n* = 159)	Reported results
Funduscopy	156 (98.1)	65 negative
		91 inconclusive
Abdominal ultrasound	154 (96.9)	94 negative
		60 inconclusive
Echocardiogram	156 (98.1)	103 negative
		51 inconclusive
		2 positive (1 IC confirmed)
Cerebral ultrasound	156 (98.1)	129 negative
		27 inconclusive
Blood cultures	154 (96.9)	66 negative
		88 positive (3 IC confirmed)
Cerebrospinal fluid	37 (23.3)	33 negative
		4 positive
Peritoneal and other sterile samples	19 (11.9)	5 negative
		14 positive (4 IC confirmed)
Catheter	18 (11.3)	4 negative
		14 positive (1 IC confirmed)
Respiratory samples	6 (3.8)	6 positive
Urine	29 (18.2)	23 negative
		6 positive (2 IC probable)

### PCR and BDG analyses.

For the analysis of sensitivity, specificity, negative predictive value (NPV), and positive predictive value (PPV), three episodes from three patients (representing three non-IC episodes) for whom the corresponding samples were not referred to the reference laboratory for PCR and BDG assessment were excluded. The only patient in whom the IC episode was caused by a Candida yeast species different from those species detectable by the multiplex PCR technique performed in the study was also excluded. As a result, 155 episodes and 122 patients were analyzed. The reference laboratory analyzed all the case and control samples that were received. PCR was performed on 667 samples (sera, blood, and sterile fluid), whereas BDG was quantified in 209 serum samples.

The results are summarized in Table 7. The PCR assay gave a positive result in blood or serum in 21.9% of cases (34/155). Of the eight IC cases analyzed, PCR gave a positive result in seven, all with C. albicans. Of the 27 PCR-positive episodes classified as non-IC episodes, 25 were cases involving C. albicans (92.6%), 1 was a case involving C. guilliermondii, and 1 was a case involving two species: C. albicans and C. tropicalis. BDG was positive in six IC episodes (with either >80 pg/ml or >120 pg/ml used as the cutoff value). Although BDG levels were markedly higher in IC episodes, there was overlap in the quantification values obtained in IC episodes and non-IC episodes. Considering the total number of samples analyzed, the diagnostic yield of biomarker detection is summarized in Table 8. The sensitivity and specificity of the PCR performed on blood/serum samples were 87.5% and 81.6%, respectively, representing a difference without statistical significance. The PPV was only 26.9%, whereas the NPV was 98.8%. The sensitivity and specificity of BDG analysis were lower (75.0% and 64.6% for a cutoff of 80 pg/ml, respectively). Similarly, the PPV was very low (14.0%), but the NPV reached 97.1%. One of the cases classified as IC probable, based on presenting signs of C. albicans infection and colonization, had a negative PCR result and low BDG quantification.

**TABLE 7:** Diagnostic performance of PCR and BDG in episodes of IC^*a*^

Variable	Value(s)			*P* value
	All episodes (*n* = 155)	IC episodes (*n* = 8)^*b*^	Non-IC episodes (*n* = 147)	
Positive PCR blood/serum/sterile fluids, %	21.9	87.5	18.4	0.00
2nd positive PCR (1 week), %	3.2	0	3.4	0.76
Positive BDG (>80 pg/ml), %	37.4	75.0	35.4	0.03
Positive BDG (>120 pg/ml), %	34.8	75.0	32.7	0.02
BDG quantification, pg/ml, median (range)	155 (0–500)	352 (0–500)	144 (0–500)	0.00

a BDG, beta-d-glucan; IC, invasive candidiasis.

b Only samples from 8 episodes of IC were available for analysis of PCR and BDG accuracy. One other episode was caused by a non-Candida yeast detected in culture and was excluded from the analysis.

**TABLE 8:** Diagnostic accuracy of PCR and BDG in all samples analyzed^*a*^

Biomarker	No. of samples (% sensitivity) (*n* = 8)	No. of samples (% specificity) (*n* = 103)	PPV (%)	NPV (%)
PCR blood/serum	7 (87.5)	84 (81.6)	26.9	98.8
Positive BDG (>80 pg/ml)	6 (75.0)	66 (64.6)	14.0	97.1
Positive BDG (>120 pg/ml)	6 (75.0)	70 (68.0)	15.4	97.2
Positive PCR or BDG	7 (87.5)	49 (47.6)	11.5	98.0
Positive PCR and BDG	6 (75.0)	89 (86.4)	30.0	97.8

a BDG, beta-d-glucan; NPV, negative predictive value; PPV, positive predictive value.

### Analysis of episodes with negative culture and biomarker assay results.

One of the objectives of this study was to evaluate whether or not detection by BDG analysis or PCR is useful in the case of negative cultures. In 27 episodes (17.4%), the cultures were negative and the PCR assay in either blood or serum was positive. The BDG levels were >80 pg/ml and >120 pg/ml in 52 and 47 episodes, respectively, corresponding to 33.5% and 30.3% of episodes with negative culture results. Either the PCR or BDG results were positive in 58 episodes (37.4%), whereas the PCR and BDG results were both positive in 19 episodes, corresponding to 12.2% of the episodes with negative culture results.

Comparisons were also made according to a positive or negative result, taking into account analyses of the same variables as those described in the preceding section. Considering the PCR results, the episodes with positive PCR were significantly associated with the presence of multiorgan failure, empirical antifungal treatment, improvement with antifungals, thrombocytopenia, and Candida species colonization (*P* < 0.01). Mortality rates were also higher in those episodes with positive PCR, but the differences did not reach statistical significance (*P* = 0.07) (Table 9). Similarly, grouping the episodes according to positive or negative results of the BDG assays using the cutoff value of >80 pg/ml, the presence of multiorgan failure and the mortality rate were associated with a positive BDG result (*P* < 0.01) (Table 10). Repeating the analysis using the cutoff value of >120 pg/ml did not change the significant variables (data not shown).

**TABLE 9:** Comparisons of PCR-positive episodes to PCR-negative episodes

Variable	Value			*P* value
	All episodes (*n* = 155)	PCR-positive episodes (*n* = 34)	PCR-negative episodes (*n* = 121)	
Death, %	12.5	23.1	9.6	0.07
Multiorgan failure, %	9.3	24.2	5.1	0.00
Empirical antifungal treatment, %	20.6	35.3	16.5	0.01
Improvement with antifungals, %	4.6	14.7	1.7	0.00
Thrombocytopenia, %	3.3	11.8	0.8	0.00
Candida colonization, %	5.3	14.7	2.5	0.02

**TABLE 10:** Comparisons of BDG-positive episodes to BDG-negative episodes using the >80 pg/ml cutoff^*a*^

Variable	Value			*P* value
	All episodes (*n* = 155)	BDG-positive episodes (*n* = 58)	BDG negative episodes (*n* = 97)	
Death, %	12.5	22.7	6.6	0.00
Multiorgan failure, %	9.3	19.6	3.2	0.00
Improvement with antifungals, %	4.6	8.8	2.1	0.05

a BDG, beta-d-glucan.

A comparison was also made with the episodes that were either PCR or BDG positive, yielding similar results, as shown in Table 11. Mortality, the presence of multiorgan failure, empirical antifungal therapy improvement with antifungals, thrombocytopenia, and Candida colonization were significantly associated with PCR or BDG positivity.

**TABLE 11:** PCR and BDG positives in IC episodes compared with non-IC episodes^*a*^

Variable	Value			*P* value
	All episodes (*n* = 155)	PCR- and BDG-positive episodes (*n* = 25)	PCR- and BDG-negative episodes (*n* = 130)	
Death, %	12.5	27.8	9.8	0.04
Multiorgan failure, %	9.3	33.3	4.7	0.00
Empirical antifungal treatment, %	20.6	40.0	16.9	0.01
Improvement with antifungals, %	4.6	16.0	2.4	0.01
Thrombocytopenia	3.3	12.0	1.6	0.03
Candida colonization, %	5.3	16.0	3.1	0.02

a BDG, beta-d-glucan.

### Multivariate analysis of IC.

Only those variables with results that were statistically significant (i.e., those for which the *P* value was <0.01) or close to significance (i.e., those for which the *P* value was <0.1) or of major clinically or epidemiologically relevance were introduced into the model. Both discriminant function analysis and multiple logistic regression analysis were performed. Due to the low number of IC cases, the statistical significance was low. As observed in the univariate analysis, IC cases were associated with statistical significance and odds ratio (OR) values of >2.0 for patients with Candida dermatitis, Candida colonization, presence of multiorgan failure, PCR positivity, and PCR and BDG positivity.

## DISCUSSION

In our study, the prevalence of IC episodes in preterm VLBW neonates was 5.7% in large referral NICU centers throughout Spain. This prevalence is similar to that reported in Spanish studies (4) and in other settings (17). It is important that no patient received antifungal prophylaxis, a practice that would probably have had an impact on the prevalence and interpretation of the diagnostic accuracy of the biomarkers. Fluconazole prophylaxis has been associated with a decrease in the incidence of IC in controlled trials in VLBW neonates (18). Although the use of fluconazole for the first weeks of life is controversial (19), fluconazole is increasingly used prophylactically in high-risk neonates in the NICU in the United States and western Europe (17, 20). This practice occurs because the signs and symptoms of IC in VLBW neonates are nonspecific and the prognosis is poor when diagnosis and therapy are delayed (1, 5). C. albicans was the most frequently isolated species in our study, as previously reported in neonates in general as well as in Spanish NICU and other settings (1, 4, 5). C. parapsilosis has been reported to be the second most commonly isolated species of Candida spp. in NICU (4, 13). No patient was diagnosed with C. parapsilosis in our series. Even with the difficulties in obtaining an accurate diagnosis of IC in VLBW neonates and the relatively small sample size, our series confirms the poor prognosis of IC with high mortality rates and rates of complications of IC episodes, underscoring the need to start early empirical antifungal therapy and to improve the microbiological diagnosis.

In our series, blood cultures had a low diagnostic yield. Only three of nine episodes of confirmed or probable IC had positive blood cultures, in accordance with the literature in neonates, in whom obtaining blood samples of sufficient volume is a major impediment to achieving an etiological diagnosis. That the diagnostic yield of blood cultures for IC in older children and immunocompromised adult patients is also low probably reflects the short duration of candidemia in cases of deep-seated candidiasis, in contrast to the more continuous release of Candida DNA that can be detected by PCR (21). Several studies have previously demonstrated that molecular techniques perform better than culture methods in both adults and children (7–12, 22). PCR has shown very promising results in immunocompromised adults and children for the diagnosis of IC, but its use in these situations requires validation. Its use in neonates is still very limited (13, 14). Taira et al. recently reported their experience with 54 neonates and children with IC in ICU, showing that PCR is a very useful tool, improving the diagnostic yield of blood cultures from 14.8% to 24% (13). A recent meta-analysis examined the yield of PCR techniques applied directly to blood samples in the diagnosis of IC, using standard PCR, nested PCR, and real-time PCR. Among control patients at risk for IC, sensitivity and specificity values were 93% and 95%, respectively, whereas the sensitivity of blood cultures in the diagnosis of IC was only 38% (7). The diagnostic yield of PCR performed using whole blood or serum is a matter of debate. Some studies have shown better performance using blood (8), whereas others have shown higher sensitivity using serum (10). In our series, the sensitivity was higher in whole blood than in serum, although the diagnostic yield was increased when the two samples were analyzed simultaneously, as reported previously (8).

Our multiplex PCR method may have important advantages over other diagnostic methods. The short turnaround time to obtaining PCR results is a major breakthrough, albeit it was not tested in our series, since all samples were stored for later analysis. In addition, this assay allows the detection of Candida spp. in small volumes of blood, which is an advantage for neonates and young children. The ability to detect infection early and discriminate Candida spp. is extremely important for planning the introduction of antifungal therapy, thus improving the outcome for infected patients and reducing the prophylactic and empirical misuse of antifungal compounds. One of the more important findings of our study is the high NPV of the PCR (98.8%), which might allow the withdrawal of unnecessary antifungal therapy, thus reducing any potential toxicity, selection of resistance, and costs. Another important advantage of PCR is the possibility of identifying several species of Candida spp., allowing the identification of those that cause bloodstream infections in these critically ill neonates, as previously shown in other pediatric studies (13). In fact, one of the episodes was positive for C. albicans and C. tropicalis simultaneously.

BDG is a component of the cell wall of most fungi that has also shown promising results for the diagnosis of IC, although there are only limited data on its use in children (15, 16). Several factors may lead to a false-positive assay result in the absence of invasive fungal infection, including serious bacterial infection, the use of dialysis membranes and filters made from cellulose, cotton gauze employed during surgery, specific fractionated blood products such as serum albumin and immunoglobulins, and the use of some antibiotics as amoxicillin-clavulanate and piperacillin-tazobactam (23). Although pertinent data were not specifically registered, none of the patients had any of the above-mentioned factors, except bacterial sepsis. Therefore, it is possible that some children with confirmed bacterial sepsis had false-positive BDG results. Colonization with Candida spp. may also be associated with high levels of BDG in children (15, 16). Serum levels may be higher in children than in adults. In addition, overlapping levels are frequently observed in children with IC and without IC, as observed in our series (15, 16). Accordingly, it is possible that some patients with negative blood cultures and negative PCR results but with positive BDG results represented false-positive diagnoses as a result of colonization.

Although the sensitivity and specificity of PCR were higher than 80%, the PPV was low due to the relatively low prevalence of Candida infection in the cohort. The performance of the BDG analysis was worse, with a sensitivity of 75% and specificity of 65% or 68% for a cutoff value of 80 or 120 pg/ml, respectively. Nevertheless, both PCR and BDG were useful in ruling out the possibility of an infection (NPVs, >97%). One of the cases classified as IC probable, based on presenting signs of C. albicans infection and colonization, had a negative PCR result and low BDG quantification, which might decrease the overall sensitivity because of the relatively small sample size. Considering only the confirmed episodes of IC, the sensitivities of PCR and BDG increased to 100% and 83%, respectively. Despite the relatively low PPV of these techniques, they may be useful in clinical practice in high-risk patients, and this is particularly true for the PCR-based technique. There is evidence to indicate that in the case of a negative culture, a positive PCR result should be considered a sign of probable infection and thus should be used as a treatment guide (8).

Another important factor to consider in the interpretation of the diagnostic yield of BDG or PCR analysis is prophylaxis with antifungal drugs. Fluconazole prophylaxis is increasingly used in NICU (17, 20) but was not used prophylactically in our study. The diagnostic accuracy of BDG or PCR analysis might be different in settings where antifungal prophylaxis is administered.

Our study had several important limitations that may limit the interpretation and comparison of the results. Although prospective in design, not all patients completely adhered to the protocol, and some patients lacked biomarker samples. Moreover, the timing of the extractions and the volume of blood drawn were not specified and may have varied. In addition, a strict definition of IC is difficult to standardize. Some clinicians consider candiduria in VLBW preterm infants to represent a true IC prompting a systemic evaluation (blood, cerebrospinal fluid [CSF], and abdominal ultrasound) for disseminated Candida infection and warranting immediate treatment (24). The two patients deemed probable IC patients had candiduria and improved with antifungal therapy, suggesting that they both had IC. A further limitation of our study was the relatively small sample size of patients with confirmed or probable episodes of IC. The lack of a sensitive and specific gold standard for the diagnosis of IC limits the interpretation of the results. Interestingly, we performed an analysis of the outcome variables in which we considered the proposed definitions of IC as well as the positivity of PCR and BDG and obtained similar results.

Despite these limitations, our study also had important strengths. It was a large prospective study that provided realistic information on the prevalence of IC in preterm VLBW neonates admitted to the NICU and on the spectrum of Candida species isolates, risk factors, and outcomes of IC in these very-high-risk pediatric patients. It also aimed to evaluate the performance of some biomarkers such as PCR or BDG for the diagnosis of IC in this patient population, providing useful information on their diagnostic yield and limitations. Even with the small number of IC episodes, this study represented one of the largest series reported so far among those that have specifically evaluated the performance of PCR and BDG analyses in VLBW neonates with suspicion of sepsis. Both biomarkers offered an attractive method for early diagnosis of IC and might be help rule out IC and minimize the inappropriate use of antifungal compounds. We conclude that PCR (using both whole blood and serum) might be a useful tool that would improve the diagnostic accuracy of IC in critically ill preterm neonates. Further studies and validation of this technique in this special population are warranted.

## MATERIALS AND METHODS

### Patients.

This was an epidemiological, noninterventional, observational case-control, nested-cohort study. It was not linked to a specific treatment. The multicenter nationwide study was undertaken in Spain and was registered and approved by the Clinical Trial and Ethics Committee of the University Hospital 12 de Octubre, Madrid, Spain (reference number CEIC 10/244). It was conducted prospectively between March 2011 and March 2013 in preterm VLBW patients in the NICU with a clinical suspicion of sepsis or meningitis. Patients were consecutively enrolled in 17 NICU centers in Spain for 2 years following inclusion of the first patient.

Inclusion criteria were as follows: survival after the first 72 h of life; very low birth weight (<1,250 g); gestational age between 24 and ≥32 weeks; and clinical suspicion of sepsis or meningitis in the NICU (cases of IC involving the central nervous system [CNS] were not distinguished from those in which it was not involved). Exclusion criteria were as follows: postnatal age of <72 h; presence of major congenital malformations; hydrops fetalis; or severe perinatal asphyxia (Apgar score at 5 min, ≤4).

Patients receiving antifungal prophylaxis were eligible to participate in the study. Empirical antifungal treatment was given according to the usual clinical practice. Blood samples for blood cultures as well as for PCR and BDG testing were collected before administration of empirical antifungal therapy. All samples were collected by venipuncture. Laboratory staff members were blind with respect to the sources of sera, blood, and sterile fluids. Samples for culture were processed immediately; the remaining samples were frozen at −80°C until tested.

Parents or caregivers gave informed consent and signatures indicating acceptance of participation in the study. The institutional review board (IRB) of one of the participating centers (University Hospital 12 de Octubre, Madrid, Spain) served as the referral center and approved the study protocol.

### Definitions.

The study population comprised VLBW preterm neonates with clinically suspected IC, sepsis, or meningitis. Cases were defined as those with a confirmed or probable diagnosis of IC according to the following definitions.

### (i) IC confirmed.

An episode was defined as IC confirmed if a culture of a sterile sample was found to be positive for Candida spp. or if a Candida species was detected during the autopsy of a deceased patient. Likewise, an episode was considered IC confirmed when endophthalmitis candidiasis was diagnosed by direct examination of the fundus of the eye or if fungus balls were observed by abdominal ultrasound.

### (ii) IC probable.

This designation was used for patients who tested negative for Candida spp. in a culture derived from blood, cerebrospinal fluid (CSF), or other sterile fluid samples but for whom (i) improvement was seen upon treatment with an antifungal(s) and/or (ii) the episode corresponded to a true IC (according to the clinician's criteria and based on the evolution) and/or (iii) the image from an echocardiogram was consistent with Candida endocarditis or a cerebral ultrasound showed microabscesses consistent with IC. In addition to fulfilling any of the criteria described above, one of the following conditions had to be met: colonization by Candida spp. was detected by isolation of Candida spp. from stool, urine, bronchial aspirate, or gastric samples or a clinical diagnosis of candida dermatitis was made; thrombocytopenia (platelet count of <100,000/mm^3^) was diagnosed; or, after an episode of sepsis, a complication such as meningitis, endocarditis, or pyelonephritis appeared as a result of the dissemination of Candida spp. This condition was determined by clinical judgment. Mucosal candidiasis in the absence of clinical signs of IC was not considered to represent IC.

The study was designed to prospectively incorporate all episodes of IC (cases) and the pool of patients with suspected sepsis or meningitis in whom IC was ruled out (controls), during the study period. The control episodes were defined as those with confirmed bacterial infection as well as those in which no microorganism was isolated and IC was ruled out.

### DNA extractions and PCR-based technique.

The PCR assay was designed to detect the six most frequent species of the genus Candida in IC in Spain (8), namely, C. albicans, C. parapsilosis, C. tropicalis, C. glabrata, C. krusei, and C. guilliermondii, using specific molecular beacon probes labeled with different fluorescent dyes. Primers and probes were designed on the basis of the nucleotide sequences of the internal transcribed spacer (ITS) ribosomal DNA region from strains belonging to the collection of the Spanish National Center of Microbiology. The probes targeted the ITS1 or ITS2 regions of ribosomal DNA. These regions were chosen as targets because they provided the opportunity to design a suitable probe for each strain. The assay consisted of two multiplex PCRs: reaction 1 (C. albicans, C. parapsilosis, and C. tropicalis), which was performed using a LightCycler Probes master kit (Roche Diagnostics, Madrid, Spain); and reaction 2 (C. glabrata, C. krusei, and C. guilliermondii), which was performed using a 2x SensiMix probe kit (Quantace, Ecogen, Madrid, Spain). The two PCRs were performed simultaneously using a LightCycler 480 system (Roche Diagnostics, Mannheim, Germany). The volume of sample for PCR was 200 μl DNA from blood and sera that was extracted using a QIAamp DNA minikit (Qiagen, Izasa, Madrid, Spain). Elution was performed with 50 μl of buffer, and the PCR was performed with 2 μl of DNA extracted from each sample. All sample analyses were performed in duplicate, and quantification standards were run in conjunction with each set of samples and negative controls (8).

BDG analysis was performed using a Fungitell assay in Fontlab 2000 (Fontlab Ltd., Barcelona, Spain) (25). Two different cutoff values (BDG of >80 pmol/ml and BDG of 120 pmol/ml) were considered representative of a positive result. Analysis was conducted separately for each cutoff value.

Clinical and laboratory variables were obtained for all patients following a preestablished protocol.

### Statistical analysis.

Statistical analysis of the results was carried out with Statistical Package for Social Sciences (SPSS) software (version 19.0; SPSS Inc., Chicago, IL, USA). Qualitative variables were expressed as absolute and relative frequencies. Quantitative data were calculated as the mean or median with range or interquartile ranges (Q1 to Q3). Categorical variables were compared using the χ^2^ test (or Fisher's exact test in cases where small numbers of samples were expected), whereas Student's *t* test or the Mann-Whitney U test was applied for continuous variables, as appropriate. Using the method of introducing significant, nearly significant, or clinically or epidemiologically interesting variables, a multivariate analysis was also performed using discriminant function analysis and multiple logistic regressions. Sensitivity, specificity, negative predictive value (NPV), and positive predictive value (PPV) were calculated for the assays, cases, and controls, and data were compared using McNemar's χ^2^ test. All statistical tests were two-tailed tests, and a strict *P* value of <0.01 was deemed significant.
